# Effect of Formula Feeding and Breastfeeding on Child Growth, Infant Mortality, and HIV Transmission in Children Born to HIV-Infected Pregnant Women Who Received Triple Antiretroviral Therapy in a Resource-Limited Setting: Data from an HIV Cohort Study in India

**DOI:** 10.5402/2012/763591

**Published:** 2012-06-03

**Authors:** Gerardo Alvarez-Uria, Manoranjan Midde, Raghavakalyan Pakam, Lakshminarayana Bachu, Praveen Kumar Naik

**Affiliations:** Department of Infectious Diseases, Rural Development Trust Hospital, Bathalapalli, Kadiri Road, Bathalapalli 515661, India

## Abstract

We describe a programme for the prevention of mother-to-child transmission (PMTCT) of HIV that provided universal antiretroviral therapy (ART) to all pregnant women regardless of the CD4 lymphocyte count and formula feeding for children with high risk of HIV transmission through breastfeeding in a district of India. The overall rate of HIV transmission was 3.7%. Although breastfeeding added a 3.1% additional risk of HIV acquisition, formula-fed infants had significantly higher risk of death compared to breastfed infants. The cumulative 12-month mortality was 9.6% for formula-fed infants versus 0.68% for breastfed infants. Anthropometric markers (weight, length/height, weight for length/height, body mass index, head circumference, mid-upper arm circumference, triceps skinfold, and subscapular skinfold) showed that formula-fed infants experience severe malnutrition during the first two months of life. We did not observe any death after rapid weaning at 5-6 months in breastfed infants. The higher-free-of HIV survival in breastfed infants and the low rate of HIV transmission found in this study support the implementation of PMTCT programmes with universal ART to all HIV-infected pregnant women and breastfeeding in order to reduce HIV transmission without increasing infant mortality in developing countries.

## 1. Background

In 2010, there were 1,490,000 HIV-infected pregnant women and 390,000 children became infected with HIV [1]. Mother-to-child transmission of HIV can occur during pregnancy, during birth, or during breastfeeding. The risk of transmission is 15–30% in nonbreastfeeding populations and breastfeeding adds an additional 5–20% risk for an overall transmission rate of 20−45% [2]. In 2010, the World Health Organization (WHO) released the guidelines on antiretroviral drugs for treating pregnant women and preventing HIV infection in infants with the goal of reducing mother-to-child transmission to less than 5% and virtually eliminating HIV infection in children by 2015 [3, 4].

Infant feeding by HIV-infected women remains a public health dilemma for developing countries. Although breastfeeding involves a considerable risk of HIV transmission, nonbreastfed infants are exposed to higher risk of death in resource-limited setting [5, 6]. According to 2010 WHO guidelines [4], National health authorities should decide whether health services will principally counsel and support HIV-infected women to either breastfeed and receive antiretroviral interventions or avoid breastfeeding, as the strategy that will most likely give infants the greatest chance of HIV-free survival. Developed countries have decided to recommend formula feeding for HIV-exposed children due to the low infant mortality and the risk of HIV transmission through breastfeeding, whereas in developing countries the health and survival benefits of breastfeeding exceed the risk of HIV transmission [7]. However, most of the evidence for recommending breastfeeding in developing countries comes from clinical trials performed in Sub-Saharan Africa. Data of formula feeding on child growth and mortality from PMTCT programmes in other continents are scarce, and some developing countries are supporting the use of formula feeding in their national PMTCT programmes [7–11].

India is the third country in the world in terms of HIV infected people, and it is estimated that 43,000 pregnant women were living with HIV in India in 2009 [12]. In this study, we investigated the effect of formula feeding and breastfeeding on mortality, growth, and HIV transmission of children enrolled in a PMTCT programme in a rural district of India. 

## 2. Methods

### 2.1. Setting

In India, the neonatal mortality rate was 34 deaths/1000 live births and the infant mortality rate was 50 deaths/1000 live births in 2009 [13]. Andhra Pradesh is the state with the highest burden of HIV-infected people in India [14]. Anantapur is a district situated in the south border of Andhra Pradesh. Rural Development Trust (RDT) is a nongovernmental organization that has three hospitals in Anantapur. In these hospitals, medical care of HIV-infected people is provided free of cost, including medicines and consultation or admission charges.

The Vicente Ferrer HIV Cohort Study (VFHCS) is an open cohort study of all HIV-infected patients who have visited the RDT hospitals since June 2006. The characteristics of the cohort have been described elsewhere [15]. Taking into account data from the Andhra Pradesh State AIDS Control Society that estimates that 15,721 HIV-infected people are living in the district of Anantapur, the cohort is fairly representative for the district as it covers approximately 70% of all HIV-infected patients [15]. For this study, HIV-infected women from the VFHCS database who became pregnant between January 1st 2008 and December 5th 2010 were included in the analysis. The selection of patients from the database was executed in January 17th 2012.

### 2.2. Description of the RDT Programme for PMTCT of HIV

In order to reduce the number of children infected by HIV, RDT initiated a programme for PMTCT. In close collaboration with governmental Integrated Counselling and Testing Centres, which provided free HIV testing to all pregnant women in the district, HIV-positive pregnant women were followed by a group of 30 community health workers spread across the district. HIV-infected pregnant women initiated triple antiretroviral therapy (ART) regardless of the CD4 lymphocyte count. Delivery by caesarean section was offered to all women for reducing the risk of HIV transmission at birth, unless they had an HIV viral load below 1000 copies/mL in the third trimester of pregnancy. In women who were diagnosed with HIV during labour, a single fixed-dose combination tablet of zidovudine 300 mg, lamivudine 150 mg, and nevirapine 200 mg was given, and caesarean section was performed only if there was no membrane rupture. Exclusive breastfeeding with rapid weaning after 5-6 months was promoted. However, women who initiated ART less than one month before delivery were counselled about the risk of HIV transmission through breastfeeding and formula feeding was offered for six months free of cost. Women who decided to give formula feeding to their babies were admitted to hospital for training on safe methods of replacement feeding such as boiling water for milk preparation and using cup feeding instead of bottle feeding [2, 16]. ART to the mother was continued after delivery if the CD4 lymphocyte count was below 350 cells/*μ*L at the moment of initiating ART. In women who initiated ART with high CD4 lymphocyte count, ART was stopped either one month after stopping breastfeeding or immediately after delivery in those women who decided to give formula feeding to their children. If the ART regiment included a nonnucleoside reverse transcriptase inhibitor (nevirapine or efavirenz), two nucleoside reverse transcriptase inhibitors were continued for two extra weeks in order to reduce the risk of developing nevirapine or efavirenz resistance due to their longer life span [4].

Newborn prophylaxis was also given. Before the publication of the WHO 2010 guidelines, single (zidovudine or nevirapine) or triple (zidovudine, lamivudine, and nevirapine) drug prophylaxis in syrup form was given to newborns for 7 to 42 days according to the risk of HIV infection during delivery. After the publication of WHO 2010 guidelines, all newborns received 28 days of zidovudine syrup according to their weight. 

For identifying the moment of HIV transmission, we aimed to perform HIV viral load at birth, after six weeks of delivery and after six weeks of stopping breastfeeding. Children were considered to be infected intrauterus, during delivery, or during breastfeeding if the HIV viral load was positive at birth, after six weeks of delivery, or six weeks after stopping breastfeeding, respectively. Children who did not have any HIV viral load determination were considered as HIV negative if their HIV serology was negative at any time after six months of age and were considered as HIV positive if they had a positive HIV serology after 18 months of age [17]. Measurement of HIV viral load was performed utilizing two different commercial assays, a real-time HIV-1 RNA polymerase chain reaction assay (Cobas TaqMan HIV-1 v1.0, Roche Diagnostics, Mannheim, Germany) until March 2011 and a reverse transcriptase activity assay (Exavir load version 3, Cavidi, Uppsala, Sweden) since March 2011. 

During the visits of children to hospitals, growth parameters such as weight, length/height, head circumference, mid-upper arm circumference, triceps skinfold thickness and subscapular skinfold thickness were collected. HIV-exposed children were followed up until 18 months of age with regular visits to the hospitals and with home visits by outreach workers.

### 2.3. Statistical Analysis

Statistical analysis was performed using Stata Statistical Software (Stata Corporation, Release 11, College Station, Texas, USA). Confidence intervals for proportions were estimated utilizing the Wilson method [18]. We used Kaplan-Meier curves and log-rank test for comparing mortality in children who received breastfeeding or formula feeding. Time was measured from birth to death or the last visit date. For calculating Z-scores of anthropometric indicators according to WHO child growth standards for age and sex, we utilized the WHO macro for Stata (igrowup_standard.ado) [19]. WHO child growth standards were calculated from a group of selected children from different parts of the world who received optimal nutrition according to WHO standards: no known health or environmental constraints to growth, mothers willing to follow specific feeding recommendations (i.e., exclusive or predominant breastfeeding for at least four months, introduction of complementary foods by the age of six months and continued partial breastfeeding up to at least 12 months), no maternal smoking before and after delivery, single term birth, and absence of significant morbidity [20]. Among all anthropometric markers, we selected severe underweight (weight-for-age < −3 Z-scores) because it has demonstrated to be a good indicator for predicting mortality in infants from India, especially in infants aged less than six months [21]. The study was approved by the ethical committee of the RDT Institutional Review Board. 

## 3. Results

During the study period, 391 HIV-infected pregnant women were identified and 336 (85.9%) entered in the PMTCT programme before delivery. Of the 55 pregnancies that did not enter in the programme before delivery, five children were lost to follow up and 12 out of 50 (24%) were HIV infected although 29 (58%) of them received formula feeding. 

Characteristics of the 336 women who entered in the programme before delivery are described in Table 1. The median age was 23 years, and the median height was 152 cm. The proportion of underweight in mothers was 39%, and approximately half of them were primipara. Almost 40% were illiterate and only one was smoker. The proportion of pregnant women with CD4 lymphocyte count below 200 and 350 cells/*μ*L were 9% and 29%, respectively. Zidovudine was used in 76% of women, and nevirapine was used more frequently than efavirenz. Near 7% initiated ART at the same day of delivery. Viral load in the third trimester was available for 199 women and 175 (88%) of them had a viral load below 1000 copies/mL. Seven percent of women delivered at home, and caesarean section was performed in 39% of the cases. Zidovudine syrup was used more commonly than other newborn prophylaxis regimens, and 51% of children received formula feeding. 

**Figure 1:**
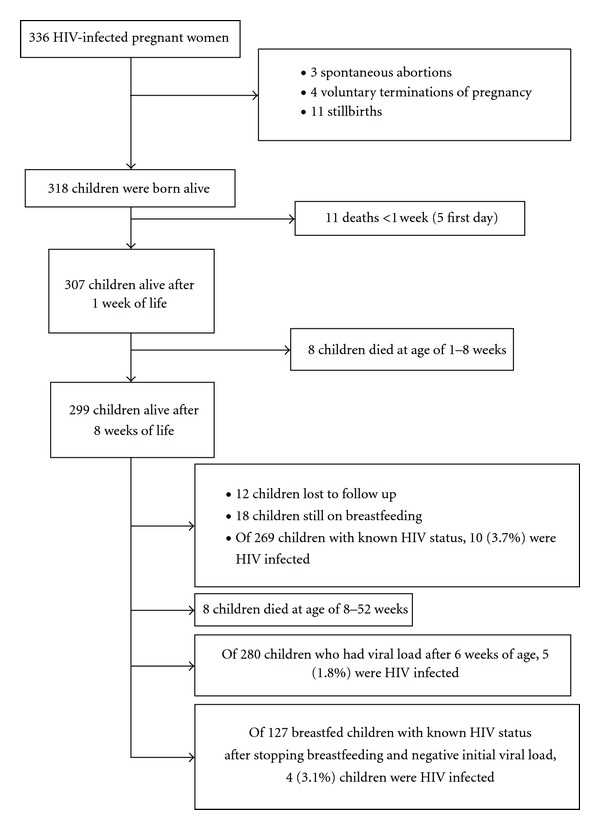
Flowchart of the outcomes of the study.

In Figure 1, we present a flow diagram of the outcomes of all HIV-positive pregnancies that entered in the PMTCT programme before delivery. Of the three pregnant women who had spontaneous abortion, two started ART (zidovudine, lamivudine, and nevirapine) before having the abortion. All voluntary terminations of pregnancy occurred between the third and the fifth month of pregnancy. Of the 11 children who died during the first week of life, weight was recorded in five and in four of them the birthweight was less than 1.5 kg. No mother died during pregnancy or during delivery. The perinatal mortality rate was 22/329 (67 deaths/1000 total births, 95% confidence interval (CI) 45 to 99), the neonatal mortality rate was 12/318 (38 deaths/1000 live births, 95% CI 22 to 65), and the infant mortality rate was 27/318 (85 deaths/1000 live births, 95% CI 59 to 121). The overall transmission of HIV was 3.7% (95% CI, 2 to 6.7), although 12 (4%) were lost to follow up and 18 who had a negative viral load after six weeks of life were still on breastfeeding at the end of the study period. Antenatal and perinatal preventive measures were able to reduce the rate of HIV transmission to 1.8% (95% CI, 0.7 to 4.1) in those children who had a viral load determination after the age of six weeks. Of those breastfed children who had a negative viral load determination after aged six weeks, 3.1% (95% CI, 1.2 to 7.8) acquired HIV during breastfeeding. In this group, ART was initiated at least one month before delivery in all but one of the cases (99.2%). The characteristics of the ten children who became HIV infected are presented in Table 2. 

**Figure 2:**
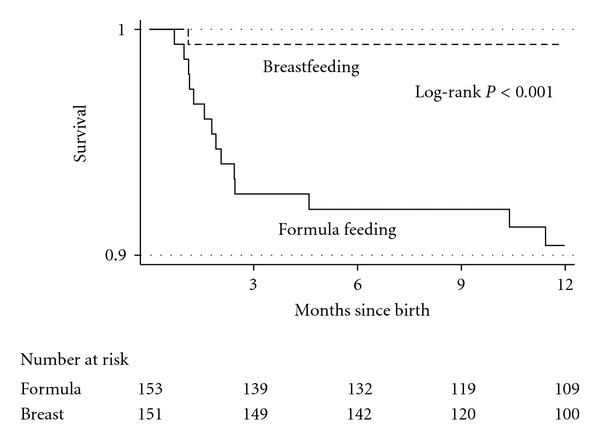
Kaplan-Meier survival curves of infants by type of feeding.

To study the effect of the type of feeding on infant mortality we excluded children who died during the first week of life to avoid causes of the death related to perinatal complications [22, 23]. Kaplan-Meier survival curves of infants by type of feeding are presented in Figure 2. Three quarters of deaths (12 out of 16) occurred during the first trimester of life. Compared to breastfed children, formula-fed children had a crude hazard ratio for death of 15.2 (95% CI, 2 to 114.8) (*P* = 0.008). In a multivariable analysis, the hazard ratio for death in formula-fed children was 13.5 (95% CI, 1.7 to 108.2) (*P* = 0.014) adjusted by low birth weight (<2.5 kg), low CD4 lymphocyte count of the mother (<200 cells/*μ*L), home delivery, illiteracy of the mother and less than one month on ART before delivery [24]. The cumulative mortality at three months was 7.3% (95% CI, 4.1 to 12.7) for formula-fed infants and 0.66% (95% CI, 0.1 to 4.6) for breastfed infants. The cumulative mortality at 12 months was 9.6% (95% CI, 6.7 to 18.5) for formula fed infants and 0.68% (95% CI, 0.1 to 4.7) for breastfed infants. Free-of-HIV survival was also significantly higher in breastfed children (126/131, 96.2%) than in formula-fed children (130/151, 86.1%) (*P* = 0.003). When questioning mothers about the symptoms of the child before death, eight reported gastrointestinal symptoms, three reported fever, three reported seizures, one reported respiratory symptoms, and one reported low consciousness. 

**Figure 3:**
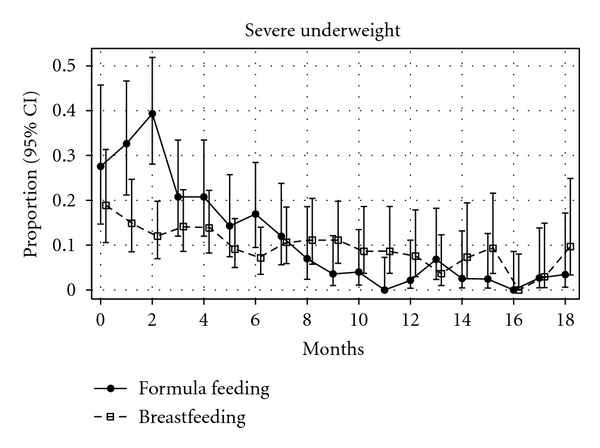
Proportion of children with severe underweight (<−3 Z-score) and 95% confidence interval during the first 18 months of life by type of feeding.

**Figure 4:**
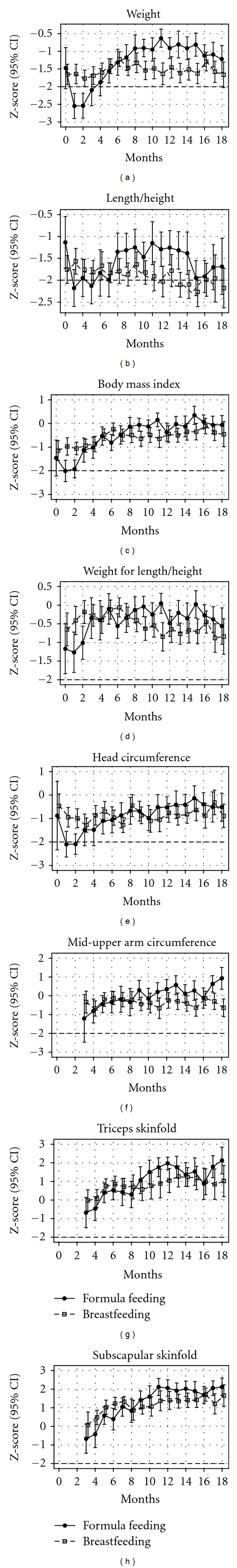
Mean and 95% confidence interval of anthropometric markers during the first 18 months of life by type of feeding adjusted by age and sex.

The proportion of children with severe underweight by type of feeding during the first 18 months of life is presented in Figure 3. The proportion of infants with severe underweight who received formula feeding was higher in the first two months of life, reaching a pick of 40% at two months, but it was reduced notably afterwards. The proportion of infants with severe underweight who received breastfeeding experienced progressive reduction over time. The mean and 95% CI of anthropometric markers (weight, length/height, body mass index, weight for length/height, head circumference, mid-upper arm circumference, triceps skinfold thickness, and subscapular skinfold thickness) for age and sex by type of feeding during the first 18 months of life are presented in Figure 4. Whereas biomarkers of breastfed infants remained more or less stable over time, formula-fed infants experienced a rapid decline during the first two months of life and a rapid improvement after the third month of life, achieving similar or superior values to breastfed infants after the ninth month of life.

## 4. Discussion

In this study, the use of formula feeding was associated with increased risk of mortality and lower HIV-free survival compared to breastfeeding. In India, 98% of children are breastfed and it is estimated that 54% of all deaths before age of five years are related to malnutrition [25]. The introduction of formula feeding for averting the transmission of HIV imposes an additional handicap to an already malnourished population. As it is shown in Figures 3 and 4, formula-fed children experience an acute decline in anthropometric markers during the first two months of life, when most of deaths occurred. Our findings are in accordance with the results of other studies performed in resource-limited settings that have shown an increased morbidity and mortality in HIV-exposed children who received formula feeding [7, 26, 27]. Also in India, HIV-exposed children from a large government hospital who were formula fed had an increased risk of hospitalization [28]. 

Although the overall proportion of HIV-infected children was 3.7%, prenatal and perinatal interventions achieved a HIV transmission rate of 1.8%, which is similar to the rate of HIV transmission in developed countries where the majority of children receive formula feeding [29]. The rate of HIV transmission due to breastfeeding was 3.1%, which is slightly higher than the rate reported in clinical trials in Africa [30, 31]. 

The breastfed infants were heavier and taller during the first two months, but lighter and shorter thereafter. Looking at the graph of weight for length/height (a marker of acute malnutrition) in Figure 4, we can observe a decline after stopping breastfeeding at six months of age compared to WHO growth standards, which were created from a population of infants who were breastfed at least until the age of 12 months [32]. However, we did not observe an increased mortality in the breastfed group after weaning at age 5-6 months as it was seen in clinical trials in Africa with abrupt weaning at the age of four months [33, 34]. This observation is important because the 2010 WHO guidelines recommend breastfeeding until the age of 12 months [4], but the initiation of mixed feeding can increase the risk of HIV transmission [24]. New studies comparing morbidity, mortality, and HIV transmission in children who receive breastfeeding for six or twelve months may clarify this matter. 

The proportion of women with underweight was similar to the proportion of women from rural areas with underweight described in the National Family Health Survey, a large-scale survey conducted in a representative sample of households throughout India [25]. In the same study, it was observed that 48% of children under five years had stunting (length/height for age < −2 Z-scores), 20% had wasting (weight for length/height < −2 Z-scores) and 43% had underweight (weight for age < −2 Z-scores). These data can explain the low values in length/height, weight for length/height and weight observed in Figure 4. However, anthropometric markers related to fat deposits such as triceps and subscapular skinfold thickness had similar or even higher values than WHO growth standards. These differences may be explained by the low quantity of proteins in the diet in poor and rural areas of India [15, 25]. 

The study has some limitations. The mother's ART was initiated at least one month before delivery in almost all children who received breastfeeding. It is possible that the rate of HIV transmission due to breastfeeding will rise if breastfeeding is encouraged to mothers who initiate ART late in pregnancy or during labour. New studies are needed to assess HIV-free survival if breastfeeding is universally recommended regardless of the time on ART before delivery. 

## 5. Conclusions

Even in rural areas of India, universal ART to HIV-infected pregnant women is able to reduce mother-to-child transmission to less than 5%. Although breastfeeding can increase the rate of HIV transmission, formula feeding produces malnutrition during the first months of life, increases mortality and, therefore, has lower HIV-free survival compared to breastfeeding. The results of this study confirm the results of clinical trials performed in Sub-Saharan Africa and support the implementation of universal ART regardless of CD4 count to all HIV-infected pregnant women and breastfeeding for all HIV-exposed children in order to achieve WHO goals of elimination of paediatric HIV without increasing infant mortality in developing countries with high levels of malnutrition.

## Figures and Tables

**Table 1:** Characteristics of patients enrolled in the programme before delivery.

	*N* (%)
Age	23.5 (21–25.2^)∗^
Height	152 (148–160)*
Underweight (BMI < 18.5)	122 (39)
First pregnancy	173 (51.49)
Education	
Higher	13 (3.88)
Secondary	128 (38.21)
Primary	48 (14.33)
No education	146 (43.58)
Smoker	1 (0.003)
CD4 lymphocyte count (cells/*μ*L)	
<200	29 (8.76)
200–350	96 (29)
350–500	102 (30.82)
>500	104 (31.42)
Time on ART before delivery	
<1 day	23 (6.91)
1–30 days	53 (15.92)
31–60 days	48 (14.41)
61–90 days	54 (16.22)
91–120 days	40 (12.01)
>120 days	115 (34.53)
ART regimen	
AZT + 3TC + NVP	148 (44.05)
AZT + 3TC + EFV	68 (20.24)
AZT + 3TC + NFV	35 (10.42)
D4T + 3TC + NVP	39 (11.61)
D4T + 3TC + EFV	19 (5.65)
D4T + 3TC + NFV	16 (4.76)
TDF + 3TC/FTC + LPVr	5 (1.49)
AZT + 3TC + LPVr	4 (1.19)
d4T + 3TC + LPVr	1 (0.3)
TDF+3TC+AZT	1 (0.3)
Home delivery	24 (7.38)
Caesarean section	125 (39.18)
Newborn prophylaxis	
AZT	229 (73.63)
AZT + 3TC + NVP	59 (18.97)
NVP	15 (4.82)
None	8 (2.57)
Type of feeding	
Formula	159 (51.29)
Breast	148 (47.74)
Mixed	3 (0.97)

*Median (interquartile range). BMI: body mass index; ART: antiretroviral treatment; AZT: zidovudine; 3TC: lamivudine; NVP, and nevirapine; EFV, efavirenz; d4T: stavudine; NFV: nelfinavir; TDF: tenofovir; FTC: emtricitabine; LPVr: lopinavir-ritonavir. Missing values for each variable were not included.

**Table 2:** Description of HIV-positive children.

No.	Education	CD4 count	Mother's ART regimen	Days on ART before delivery	Caesarean section	Primipara	Institutional delivery	NBP regimen	Days on NBP	Birth weight (kg)	Gender	Feeding type	VL at birth	VL at 6–8 weeks	Type of HIV transmission	Comments
1	Secondary	774	AZT + 3TC + EFV	1	No	Yes	Yes	AZT	28	2.9	Female	Formula	Negative	Positive	Delivery	
2	Secondary	202	AZT + 3TC + NVP	1	Yes	Yes	Yes	AZT	28	2.07	Male	Formula	Positive	Positive	IU	
3	No education	144	AZT + 3TC + NVP	12	No	Yes	Yes	NVP	1	—	Female	Formula	Not done	Not done	IU/delivery	
4	No education	359	AZT + 3TC + NFV	70	Yes	Yes	Yes	AZT	7	2.25	Female	Breast	Not done	Positive	IU/delivery	
5	No education	251	D4T + 3TC + EFV	71	No	No	Yes	NVP	1	—	Female	Mixed	Not done	Negative	Breastfeeding	Poor adherence
6	Higher	306	AZT + 3TC + NFV	83	No	Yes	Yes	AZT	7	2.75	Female	Formula	Not done	Positive	IU/delivery	
7	Primary	410	AZT + 3TC + NFV	88	No	No	Yes	AZT	7	2.1	Female	Breast	Not done	Negative	Breastfeeding	
8	Secondary	402	AZT + 3TC + NVP	91	No	Yes	Yes	AZT	7	2.2	Female	Breast	Not done	Negative	Breastfeeding	
9	No education	273	AZT + 3TC + NVP	95	Yes	No	Yes	AZT	7	3.25	Female	Mixed	Not done	Negative	Breastfeeding	
10	Unknown	299	AZT + 3TC + NVP	356	No	No	No	AZT + 3TC + NVP	42	2	Female	Formula	Not done	Positive	IU/delivery	Poor adherence

ART: antiretroviral treatment; NBP: newborn prophylaxis; VL: viral load; AZT: zidovudine; 3TC: lamivudine; NVP: nevirapine; EFV: efavirenz; d4T, stavudine; NFV: nelfinavir; IU: intrauterus.
